# P2X receptors: dawn of the post-structure era

**DOI:** 10.1016/j.tibs.2009.09.006

**Published:** 2010-02

**Authors:** Mark T. Young

**Affiliations:** School of Biosciences, Cardiff University, Museum Avenue, Cardiff, CF10 3AX, United Kingdom

## Abstract

P2X receptors are non-selective cation channels gated by extracellular ATP. They play key roles in various physiological processes such as nerve transmission, pain sensation and the response to inflammation, making them attractive drug targets for the treatment of inflammatory pain. The recent report of the three-dimensional (3D) crystal structure of zebrafish P2X4.1 represents a step change in our understanding of these membrane ion channels, where previously only low-resolution structural data and inferences from indirect structure–function studies were available. The purpose of this review is to place previous work within the context of the new 3D structure, and to summarize the key questions and challenges which await P2X researchers as we move into the post-structure era.

## Purinergic signalling and P2X receptors

The well-established field of purinergic signalling investigates the effects of extracellular adenosine and adenine nucleotides [1,2]. Purinergic receptors are classified into three families: adenosine receptors (P1), ATP-gated ion channels (P2X) and G-protein coupled receptors (P2Y). ATP is released from synapses as a neurotransmitter, is present at high local concentrations at sites of injury and cell damage, and is also released by cells under mechanical stress *via* as yet unknown mechanisms. The released ATP acts directly on P2X and P2Y receptors, and is also converted to ADP (which also acts on some P2Y receptors), AMP and adenosine by multiple families of ecto-ATPases at the cell surface [3]. The physiological effects of extracellular purines are manifold; P2X receptors in particular play key roles in synaptic transmission [4], taste sensation [5] and control of smooth muscle [6,7]. The subtypes P2X4 and P2X7 are also critically involved in pain and inflammation (Box 1), making them of particular interest to drug companies as targets for analgesic and anti-inflammatory therapies.

P2X receptors are widely expressed in many tissues, and have been cloned from various eukaryotic organisms, including mammals [8,9], zebrafish (*Danio rerio*) [10], flatworms (*Schistosoma mansoni*) [11], slime mould (*Dictyostelium discoideum*) [12], green algae (*Ostreococcus tauri*) [13] and water bears (*Hypsibius dujardini*) [14]. However, they do not appear to be present in the genomes of *Drosophila melanogaster, Caenorhabditis elegans*, yeast or prokaryotes, making their evolutionary origins something of a mystery [15].

There are seven P2X receptor subtypes in mammals, termed P2X1 through P2X7. Functionally, P2X receptors are non-selective ligand-gated cation channels. Upon binding ATP, they permit the passage of cations along their electrochemical gradients; in cells this leads to the influx of sodium and calcium. This in turn leads to depolarization of the cell and downstream calcium signalling. The ATP sensitivity and functional properties of P2X receptors vary widely; for example, P2X1 is activated by nanomolar concentrations of ATP, and desensitizes rapidly, whereas P2X7 requires high-micromolar concentrations of ATP, and does not appear to desensitize [16]. Non-physiological agonists such as (2′-3′)-benzoyl-benzoyl ATP (BzATP) are more potent than ATP at P2X7 receptors; and more potent at rat P2X7 than mouse P2X7 [17]. Furthermore, P2X receptors display differential sensitivity to divalent cations; for example, zinc potentiates ATP-evoked currents at rat P2X2, but inhibits them at rat P2X7 [16].

In terms of their subunit arrangement and topology, P2X receptors are distinct from other ligand-gated ion channels. They are trimers [18,19], with two transmembrane (TM) domains per monomer and large (∼270 amino acids), glycosylated extracellular domains. This topology is similar to that of the acid-sensing ion channel family (ASIC) [20,21], but P2X receptors and ASICs do not share amino-acid similarity.

Until very recently, progress towards understanding the structure of P2X receptors was slow, relying on low-resolution studies and indirect structure–function experiments. However, recent publication of the three-dimensional (3D) structure of zebrafish P2X4.1 at a resolution of 3.1 Å [22] represents the greatest advance in this field since the cloning of the first P2X receptors in 1994.

The main aims of this review are to analyze the new crystal structure within the context of key recent structural and structure–function experiments, assess its significance to the P2X research community, and outline the key questions which remain unanswered about this important family of transmembrane ion channels.

## The first P2X receptor structure

Kawate and colleagues reported the crystal structure of a truncated mutant of the zebrafish P2X4.1 receptor (ΔzfP2X4.1) [22]. 26- and 8-residue segments were removed from the extreme N- and C-termini, respectively, to generate a construct which crystallized and diffracted to a resolution of 3.5 Å. Three further amino-acid substitutions (C51F, N78K and N187R) were introduced into this construct to reduce non-native disulfide bond formation and N-glycosylation, yielding crystals that diffracted to 3.1-Å resolution. It is not uncommon for such extensive mutagenesis to be required to render membrane proteins suitable for 3D crystallization; membrane proteins are notoriously difficult to work with, and this project was no different, reportedly requiring seven years to come to fruition [23]. Determining the effects of the mutagenesis on protein function is important. In this case, both constructs retained some level of ATP-gated channel function when expressed in mammalian cells; however, it is apparent that receptor function was changed [22]. Agonist sensitivity was increased as compared with wild type, whereas maximum currents appeared to be greatly reduced. The crystal structure will be different from the structure of wild-type zfP2X4.1 because it was derived from a mutant construct and the mutations had a significant effect on ion channel function. However, because the ΔzfP2X4.1 construct retained some ion channel function, it is a fairly safe assumption that structural differences between the mutant construct and the wild-type receptor will be relatively minor.

**Figure 1:**
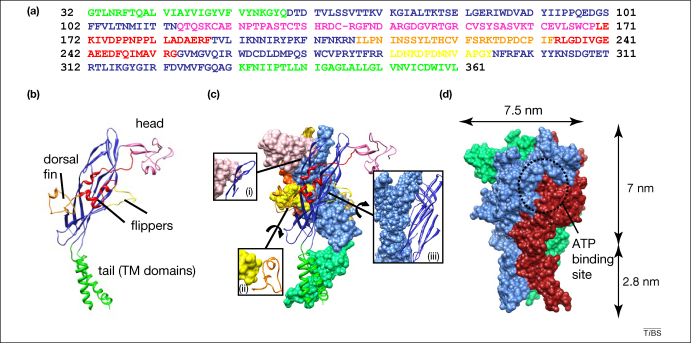
Representations of the ΔzfP2X4.1 structure. The first P2X receptor structure provides a wealth of new information about the overall fold of the molecule, subunit–subunit interactions, and hints at the location of the ATP binding site. Regions of amino acid sequence **(a)** contributing to each domain within the ΔzfP2X4.1 crystal structure are shown in the same colors on a ribbon representation of a single subunit **(b)**. The main chain electron density for residue Lys^136^ was unclear; this residue is shown as a dashed line. The subunit structure resembles a ‘leaping dolphin’; TM domains (tail; green), head (pink), dorsal fin (orange), left flipper (yellow) and right flipper (red) are indicated. **(c)** Sites of subunit–subunit interaction. Two subunits are shown, one (front) in ribbon representation and one (back) in space-filling representation. Panels depict sites of significant interaction; head–body (i), left flipper–dorsal fin (ii), and body–body (iii). The direction of rotation of the structure to obtain the views in panels (ii) and (iii) is indicated with curved arrows. **(d)** Space-filling representation of the trimer; individual subunits are colored red, blue and green. The putative ATP-binding pocket is indicated, as are the dimensions of the extracellular and TM-domains. Figure partly redrawn from [22], with permission.

Regions of the ΔzfP2X4.1 protein sequence can be related to domains within the crystal structure by imagining the monomer using the analogy of a leaping dolphin [22] (Figure 1a, b). The body of the dolphin is a β-sandwich domain which makes extensive subunit–subunit contacts in the upper regions (Figure 1c); however there are relatively few contacts in the regions proximal to the TM domains. This conformation might allow the TM domains the latitude to move upon agonist binding [22]. When the atoms within the structure are shown in space-filling representation, the dolphin-like monomer subunits are entwined round one another, and cavities between subunits thought to be the ATP binding sites are clearly visible (Figure 1d). There is no immediately obvious pathway for ions through the TM domains, consistent with the expectation that the crystallized protein is in a closed state.

## What the structure tells us about P2X biology

The ΔzfP2X4.1 crystal structure shows the overall fold of the closed state of the ion channel, confirming that P2X receptors are trimers, and showing that the TM domains are α-helical. In addition, the huge amount of detail within the structure permits the analysis and interpretation of previous work, in particular the large body of mutagenesis-based structure-function studies. It is impressive to note the high level of accuracy in the conclusions drawn from these studies, given that they were made in the absence of good homology models or structural data. Selected examples of the accuracy of well-designed structure–function experiments are given below.

### Disulfide bonds

**Figure 2:**
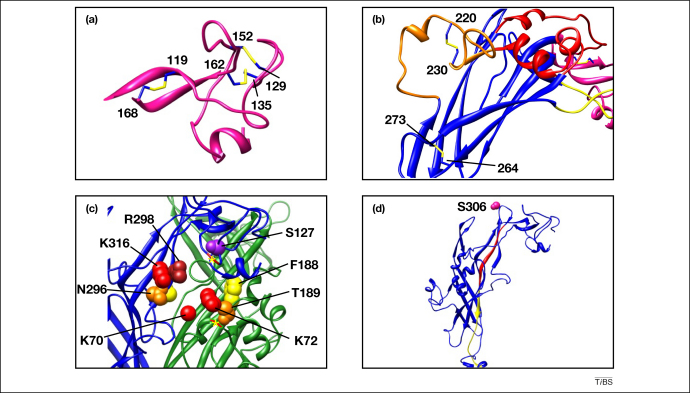
Structural interpretation of structure–function data. Previous structure–function studies, done in the absence of structural information, fit very well with the ΔzfP2X4.1structure. **(a)** Close-up of the head domain (pink sequence in Figure 1a) demonstrating that the three disulfide bonds within this region form the arrangement predicted [24,25]. Disulfides are shown in yellow and each cysteine is labelled with its corresponding residue number in the zfP2X4.1 sequence. **(b)** Close-up of the body and dorsal fin regions of the ΔzfP2X4.1structure (shown in blue and orange, respectively), showing the positions of the remaining two disulfide bonds (yellow) in the extracellular domain. **(c)** Close-up of the putative ATP binding cavity formed between two subunits. Key residues predicted to be involved in ATP binding from structure–function experiments are indicated; Phe^297^ is not labelled but is shown in yellow behind Asn^296^ and Arg^298^. The positions of residues which form part of an inter-subunit zinc binding site in rat P2X2 [31] are indicated by yellow asterisks (His^219^, bottom; Pro^125^, top). Ser^127^, a residue in the head domain close to the ATP binding site which corresponds to Lys^127^ in rat P2X7 (thought to be involved in BzATP binding; [17]), is shown in purple. **(d)** Side-view of one subunit showing the pre-TM2 region, which is critical for ion channel gating. Ser^306^, which corresponds to the glycosylated residue Asn^298^ in rat P2X2, is shown in pink at the top of the structure. The region of pre-TM2 which caused most disruption to channel glycosylation and function in cysteine mutagenesis experiments [40] is shown in red, whereas the region which tolerated cysteine mutants well is shown in yellow. Both regions are part of the same β-strand; however, the red section appears to make more extensive contacts to other parts of the structure than the yellow section, giving a sensible interpretation to the experimental data.

All mammalian P2X receptors contain ten conserved cysteine residues in their extracellular domains, which are thought to form disulfide bond pairs. If we number the cysteines from 1 to 10, from the N-terminus to the C-terminus, the cysteines were previously predicted to be paired in the order 1–6 (Cys^119^–Cys^168^; zfP2X4 numbering), 2–4 (Cys^129^–Cys^152^), 3–5 (Cys^135^–Cys^162^), 7–8 (Cys^220^–Cys^230^) and 9–10 (Cys^264^–Cys^273^) [24,25]. This pairing pattern was confirmed in the ΔzfP2X4 structure [22] (Figure 2a, b). Disulfide pairs 1–6, 2–4 and 3–5 are all located within the head region of the structure (Figure 2a), whereas disulfide pair 7–8 is located in the dorsal fin region, and disulfide pair 9–10 is located towards the TM domains at the bottom of the body region (Figure 2b). Interestingly, the two disulfide bonds composed of cysteines 2–4 and 3–5 are very close together in space, raising the possibility that these bonds could exchange with each other during and after biosynthesis.

### Agonist binding site

P2X receptors do not contain canonical ATP-binding domains such as Walker motifs, and the nature of the ATP binding site has been subject to much speculation and experiment. Based upon mutagenesis data and binding studies, eight highly conserved residues have been proposed to be involved in ATP binding. In zfP2X4.1 these are Lys^70^, Lys^72^, Phe^188^, Thr^189^, Asn^296^, Phe^297^, Arg^298^ and Lys^316^ [26–29]. The exact nature of the interaction of these residues with ATP is poorly understood; the positively charged residues Lys^70^, Lys^72^ and Lys^316^ might be involved in coordinating the negatively charged triphosphate moiety [26], whereas Phe^188^, Thr^189^, Asn^296^, Phe^297^ and Arg^298^ might be involved in binding to the adenine ring and ribose moiety [29]. Although the structure of ΔzfP2X4.1 was solved in the absence of ATP, these residues are observed to line a cavity which forms between subunits in the extracellular domain, strongly suggesting that this is the location of the ATP binding site [22] (Figure 2c). Such an inter-subunit ATP binding site is also consistent with recent mutagenesis and crosslinking studies [27,30]. The putative ATP binding pocket is surrounded by the ‘head’ and ‘left flipper’ domains of one subunit, and the ‘dorsal fin’ domains of another, so it is natural to suggest that aminoacid differences in these regions might account for the differential sensitivities of different P2X receptor subtypes to divalent cations and agonists. By example, the rat P2X2 receptor contains an inter-subunit zinc-binding site (rat P2X2 activity is enhanced by zinc), contributed to by the residues His^120^ and His^231^ (corresponding to Pro^125^ and His^219^ in zfP2X4.1) [31]. In the ΔzfP2X4.1 structure these residues are located in the head and dorsal fin regions, very close to the putative ATP binding site (Figure 2c). Another mutagenesis study suggested that the increased potency of BzATP at rat P2X7 compared with mouse P2X7 was mostly due to a single amino acid change (Lys^127^ in rat to Ala^127^ in mouse) [17]. Interestingly, the corresponding residue in zfP2X4.1 (Ser^127^) is positioned within the ‘head’ region, very close to the putative ATP binding site (Figure 2c), lending weight to the hypothesis that the benzoyl-benzoyl group of BzATP might be involved in binding this residue. A final illustration derives from a study by Adriouch *et al*, demonstrating that, in mouse P2X7, the residue Arg^125^ could be ADP-ribosylated, leading to irreversible receptor activation [32]. This residue corresponds to Pro^125^ in the head region of zfP2X4.1 (Figure 2c), and is implicated in zinc binding to rat P2X2, very near to the putative ATP binding site. The structural interpretation of these data is that the binding sites for ATP and ADP-ribose are in very similar positions, exactly as proposed by Adriouch *et al*. [32].

### TM domains and the pore region

There is strong evidence from cysteine mutagenesis and functional studies to suggest that the second TM (TM2) has the major role in ion transduction, and that the channel pore narrows significantly toward the middle of the TM domains [33–37]. The ΔzfP2X4.1 structure confirms these results; the TM domains are highly tilted, with the three TM2 subunits framing the pore, supported from the outside by the three TM1 subunits [22]. In the closed ΔzfP2X4.1 structure the channel is blocked by predominantly hydrophobic residues for approximately 8 Å of its length. Ala^344^ defines the narrowest section of the putative pore gate; this residue corresponds to Thr^336^ in rat P2X2, which has been proposed to be part of the narrowest part of the pore in that channel subtype [35]. In summary, the architecture of the pore in the ΔzfP2X4.1 structure agrees very well with structure–function data, providing further evidence that the structure accurately reflects that of the wild-type, closed channel. The architecture of the P2X receptor pore is very similar to that of the functional, desensitized chicken ASIC1 structure [20]. This is surprising because the two receptors share no amino acid-sequence similarity, and their 3D structures are very different. Whether or not P2X receptors and ASICs are a result of divergent evolution from one common ancestor, or convergent evolution from two different proteins, remains to be elucidated.

### Signal transduction module

The region immediately N-terminal to TM2 (Arg^304^-Ile^328^; rat P2X2 numbering) is highly conserved in P2X receptors, and is critically involved in the transduction of ATP binding to channel gating [38–40]. The ΔzfP2X4.1 structure showed that this region is a β-strand that runs nearly the full length of the extracellular domain [22] (Figure 2d). The upper-half passes next to the putative ATP binding site, where Lys^316^ (Lys^308^ in rat P2X2), a residue implicated in ATP binding and gating [28,41], is located. The lower-half of the pre-TM2 region makes fewer contacts with neighboring residues as it descends towards TM2. A recent structure–function study [40] showed that only amino acid substitutions within the upper-half of the pre-TM2 region in rat P2X2 (Arg^304^-Ile^314^; rat P2X2 numbering; Figure 2d) caused a change in the conformation of the protein, which was observed as alterations in N-linked glycosylation at Asn^298^ (the most proximal N-glycosylation site). Although this N-glycan site is not conserved in the ΔzfP2X4.1 structure, the corresponding region in the sequence alignment (Ser^306^) is exposed at the top of the structure (Figure 2d). By using the structure, a sensible interpretation of these data can be provided; the upper-half of TM2 appears to be involved in key protein–protein interactions, disruption of which will disrupt the structure of the receptor. There are fewer interactions in the lower-half of the pre-TM2 region (Figure 1c), making it likely that point mutants within this region would be less disruptive to the receptor structure. This interpretation is supported by the work of Roberts and Evans [38], who found that mutants within the lower-half of the pre-TM2 region (Asp^315^-Ile^328^; rat P2X2 numbering) had little effect on the function of rat P2X1, and Duckwitz *et al.* [42], who found that alanine substitution in the lower-half of the pre-TM2 region of a full-length human P2X5 construct had relatively little effect on channel function.

The sensitivity of the upper-half of the pre-TM2 region to mutation, coupled with the fact that it contains a key residue involved in ATP binding (Lys^316^; Lys^308^ in rat P2X2), demonstrates the importance of this region of the protein in ion-channel function. It is tempting to speculate that the binding of ATP induces a conformational change in the receptor structure which leads to a vertical movement of the pre-TM2 region, causing TM2 to move relative to TM1 and open the channel pore.

### The future of P2X receptor structure–function studies

The ΔzfP2X4.1 structure has enabled analysis and interpretation of previous structure–function work. In addition, it will enable highly focused structure–function studies using homology models of other P2X receptor subtypes from different species. Data derived from these studies will be more readily interpreted, and so it is expected that there will be a significant increase in the amount and effect of P2X receptor structure–function studies as a result of publication of the first crystal structure of a P2X receptor.

## Comparison of molecular models and low resolution structures

The availability of a crystal structure permits assessment of the accuracy of existing structural data for P2X receptors, derived from computer modeling, atomic force microscopy (AFM), and transmission electron microscopy. Before publication of the ΔzfP2X4.1 crystal structure at 3.1-Å resolution, the highest resolution data obtained for P2X receptors was approximately 20 Å [43,44]. Although much less detail is present in low-resolution structure studies, the conclusions are important for three main reasons: (i) the studies are done on full-length, wild-type receptors; (ii) they often provide estimates of overall size, shape and particle volume which can be compared with high-resolution data; and (iii) in some studies it has been possible to record low-resolution data in the presence of ATP, enabling the imaging of open channels.

### Molecular models

Several molecular models have been proposed for part or all of the P2X receptor sequence. Based on experimental evidence from mutagenesis experiments, a 3D model of conserved amino acids proposed to contribute directly to ATP binding was generated [45]. With little other information to draw upon, it is not surprising that the relative positions of key residues are significantly different than in the crystal structure. For example, the residues corresponding to Phe^188^ and Phe^297^ are adjacent in the model [45], but on opposite sides of the putative ATP binding pocket in the structure [22] (Figure 2c).

Three further models of P2X receptors have been described; all rely to some degree upon prediction of secondary structure. A model of P2X3, constructed using a combination of secondary structure prediction and structure–function data, proposed the existence of four putative ATP binding sites within a single subunit [46]. The possibility of there being more than one ATP binding site per monomer cannot be ruled out at present, but none of the putative ATP binding sites resembles any part of the ΔzfP2X4.1 crystal structure. A model of part of the extracellular domain of rat P2X4 (residues 180–326), based upon secondary structure similarity with aminoacyl-tRNA synthetases, was constructed [47] and an intra-subunit ATP binding site proposed based upon mutagenesis studies. This shares no significant similarity with the ΔzfP2X4.1 crystal structure. The final model was of rat P2X2, based upon a manually constructed secondary structure alignment with the ASIC using the chick ΔASIC1 structure [21] as a template [48]. This model also shares little similarity with the ΔzfP2X4.1 structure because, as we know now, P2X and ASIC possess very different secondary structures and folds, although they do share striking similarities in the shape of their transmembrane domains and pore architecture [20]. In conclusion, the accuracy of the molecular models has suffered because of the absence of suitable templates for homology modeling. However, now that we have a crystal structure to work from, future models of other P2X receptor subtypes should be more accurate, providing a wealth of testable hypotheses.

### AFM

Direct structural studies were carried out on rat P2X2 and rat P2X4 using AFM. This technique provides low-resolution images of protein surfaces, and the sample mounting technique can cause dehydration of biological specimens; nevertheless, it can give impressive results. Nakazawa *et al.* purified rat P2X2 over-expressed in insect cells and imaged particles of approximately 10 nm in diameter in the presence of ATP, and saw what appeared to be a central depression indicative of a pore [49]. Barrera *et al.* carried out elegant studies on rat P2X2 purified from over-expression in human cells using antibodies against the C-termini of the receptors, measuring an average angle of 120° between antibodies on doubly labelled particles [18]. In this manner they provided evidence of the trimeric nature of P2X receptors. They also measured the molecular volume of the imaged particles, finding it to be 409 nm^3^ on average. This volume includes one fully glycosylated rat P2X2 trimer (180 kDa), and a detergent micelle (approximate mass, 60 kDa), which is needed to protect the hydrophobic TM domains from the aqueous solvent during protein purification. Dividing the volume by the total mass gives a value of 1.7 nm^3^/kDa, which agrees reasonably well with the value of 1.2 nm^3^/kDa for a ΔzfP2X4.1 trimer (mass 125 kDa, volume 155 nm^3^), estimated from the crystal structure [22].

More recently, Shinozaki *et al.* used fast-scanning AFM and single-particle averaging techniques to image rat P2X4 trimers on a polylysine-coated mica substrate [50]. They showed that, in the absence of ATP, receptors appeared as rounded triangles when viewed from the top, having an approximate mean diameter of 12.6 nm, which agrees well with previous data [49]. Strikingly, after several minutes of treatment with 1 mM ATP, significant structural changes were observed; the rounded triangle separated into three discrete circular densities, which the authors concluded was likely to indicate each subunit moving away from a central pore as the channel opened. Although the mean particle diameter was in close agreement with previous studies, the mean particle height was <4 nm, much shorter than the length of the ΔzfP2X4.1 structure (approximately 10 nm; [22] Figure 1d). This discrepancy persisted when channels were imaged in lipid bilayers, and might have resulted from the channels being significantly tilted or bent [50].

### Electron microscopy (EM)

EM and single-particle analysis (SPA) are powerful techniques for the study of protein structure. Many thousands of individual protein molecules are imaged, classified according to their two-dimensional (2D) orientation on the grid, and then combined in 3D space to produce a structure. The simplest way to image proteins is by using a negative stain such as uranyl acetate, which stains all areas except where protein is present. The advantage of negative stain is that it gives high-contrast images, but the disadvantages are that: (i) resolution is limited to approximately 20 Å and (ii), for membrane proteins, the detergent micelle which protects the TM domains is not penetrated by the stain, precluding observation of the TM domain architecture. (This is because the protein location and orientation is inferred from the stain-excluded areas, rather than by directly imaging the protein itself.)

To obtain information at higher resolution, it is necessary to employ cryo-EM techniques. Sample damage is minimized by recording at very low temperatures, and samples must be frozen very quickly to prevent formation of crystalline ice. This technique gives very low-contrast images but, on large particles, preferably with a high degree of symmetry, can give resolutions of <10 Å. A significant additional advantage is that the protein molecule is directly imaged, allowing visualization of the TM domains. However, due to technical limitations and sample damage problems, the practical size limit for cryo-EM is ∼200 kDa [51].

Recently, Mio *et al.* generated a structure of rat P2X2, purified from over-expression in insect cells, using EM and SPA, first in negative stain, and then under cryo-conditions [52,43]. Unfortunately, due to uneven staining, the authors could not carry out 3D reconstruction in negative stain, but they could provide a cartoon representation of the trimer, with dimensions consistent with a molecule of volume ∼1200 nm^3^. The molecular mass one rat P2X2 trimer plus a detergent micelle is approximately 240 kDa, and dividing the volume by the molecular mass gives a value of ∼5 nm^3^/kDa, which is much higher than that recorded for rat P2X2 by AFM or the ΔzfP2X4.1 crystal structure (1.7 and 1.2 nm^3^/kDa respectively; see above). The authors stated that they observed a large, stain-permeable cavity within the molecule, but did not assign its orientation [52]. A later study by the same research team made use of cryo-conditions, where the unstained protein molecules were embedded in vitreous ice [43]. Rat P2X2 receptor trimers are just below the practical mass cut-off for cryo-EM (200 kDa), so it is unsurprising to find that, even though >90000 automatically selected particles were used in the reconstruction, a resolution of only 22 Å was achieved (as measured by standard criteria) [43]. The final cryo-EM structure of rat P2X2 shows two major discrepancies versus the ΔzfP2X4.1 crystal structure. First, as for the negative stain structure, the cryo-EM structure is much bigger; it has dimensions of 202 × 160 Å, compared with 100 × 75 Å for the crystal structure [22]. Second, the cryo-EM structure has several large cavities running through it, at positions inconsistent with those observed within the crystal structure. The authors state that the volume enclosed by the protein density within the cryo-EM structure is approximately 390 nm^3^, and encloses a mass of 324.5 kDa. Although the protein volume is consistent with that obtained for rat P2X2 by AFM [18], the mass of protein within that volume is almost a factor of two greater than the mass of one P2X2 trimer. It would be interesting to see the volume for this protein structure displayed at a threshold consistent with a mass of 180 kDa, but it is likely that the density would be even more disjointed. A simple interpretation of these data is that the single particles imaged by EM were small, heterogeneous aggregates of rat P2X2 trimers. Partial aggregation of rat P2X4 has been observed using EM [22]. Averaging and reconstruction of small heterogeneous aggregates would also be likely to lead to a final structure with significant internal cavities.

**Figure 3:**
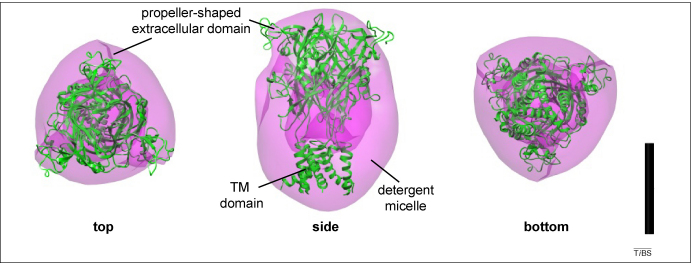
The ΔzfP2X4.1 structure fits well within a low-resolution model of human P2X4. Top, side and bottom views of a manual fit of the ΔzfP2X4.1 trimer (green ribbons) within the isosurface volume representation of human P2X4 (semi-transparent pink density) derived from electron microscopic analysis of purified single protein particles [44]. The extracellular domain fit is quite good, but the TM domains in the single particle structure are surrounded by a detergent micelle, so they appear rounded and do not fit well with the TM region of the crystal structure. The overall particle dimensions are consistent with those of the crystal structure. Scale bar = 5 nm.

EM studies of human P2X4 purified from over-expression in human cells [44] yielded a structure at 21-Å resolution which was more consistent with the recent ΔzfP2X4.1 crystal structure. In this case, trimers were selectively purified using non-denaturing gel electrophoresis, and the particles imaged in negative stain. Domain-specific labelling was used to correctly identify the propeller-shaped side of the molecule as the extracellular domain (Figure 3). The dodecyl maltoside (detergent) micelle probably explains the bulbous shape present at the other end of the molecule (Figure 3). Nonetheless, the approximate dimensions of the structure (12-nm long × 8-nm wide) agree well with those of the ΔzfP2X4.1 crystal structure. The molecular volume of the structure was 270 nm^3^, which includes one human P2X4 trimer (165 kDa) and one 60-kDa detergent micelle. Dividing the volume by the molecular mass gives a value of 1.2 nm^3^/kDa, which is entirely consistent with the value obtained for the ΔzfP2X4.1 crystal structure (also 1.2 nm^3^/kDa). In addition, the ΔzfP2X4.1 crystal structure fits very well into the low-resolution human P2X4 structure (Figure 3), and the fit of the extracellular domain is particularly good.

### The future of low-resolution studies

The advantage of low-resolution structural studies is that the full-length, wild-type protein can often be imaged in a near-native environment; however, the drawback is that the achieved resolution is often much lower. In addition, P2X receptors are below the practical size limit for high-resolution cryo-EM and SPA. However, sub-nanometer resolution could readily be achieved if the purified protein could be induced to form 2D crystals upon reconstitution into a lipid bilayer; this will presumably be a focus of future efforts with human P2X4. A study of this nature would also shed light on the dimensions and orientation of the TM domains within the more physiological environment of a lipid bilayer.

## It is all in the expression

For those of us involved in studying P2X receptor structure, it is important to know how we might obtain the highest yield of active protein for our experiments. The answer seems clear: the baculovirus system can produce protein of sufficient quality for 3D crystallography [22] as well as functional P2X receptors [49,52,43,53,54]. Mammalian [18,44,53] cell systems also have been used to express P2X receptors for structural and functional studies; it remains to be seen whether or not expression in mammalian cells can lead to high-resolution membrane protein structures.

Also of key importance is the purification of stable, trimeric forms of P2X receptors. Kawate *et al.* demonstrated the power of fluorescence-based size-exclusion chromatography in pre-screening of P2X receptor isoforms to select the most stable trimers [22,55]. Pure trimers of P2X receptors can also be isolated by electrophoresis in the presence of the non-denaturing detergent perfluorooctanoic acid [44]. The relative proportion of trimers varies with P2X receptor subtype; for example, *Dictyostelium* P2XA trimers appear to be more stable than those of human P2X4 [12]. The factors governing subunit–subunit association and trimer stability remain to be elucidated, but could prove important in designing stable constructs of other P2X receptor subtypes for structure study.

## Concluding remarks

The recent report of the first crystal structure of a P2X receptor [22] represents a major advance in our understanding of this unique and important family of ligand-gated ion channels. This work has enabled the interpretation of a large body of structure–function work, and assessment of the accuracy of recent low-resolution structure studies. It also highlights the great deal of work that is still to be done (Box 2). This achievement serves as great encouragement to others working within the field of membrane protein structural biology; membrane proteins are not impossible to work with and, with substantial effort, their structures can be revealed. We hopefully will not have to wait long before other P2X structures are available, from different subtypes and in different activation states, enabling elucidation of the mechanism of this important and fascinating family of ion channels.

## Box 1 P2X receptors in pain and inflammation

P2X receptors are important drug targets primarily because of their involvement in pain following nerve damage and inflammation. It has been known for several years that ATP injection into the skin of human volunteers causes pain, but the mechanisms governing this sensation are unclear [56]. In a rat model of neuropathic pain (tactile allodynia; animals become hypersensitive to pain after peripheral nerve injury), Tsuda *et al.* found that knocking down levels of P2X4 via antisense oligonucleotides decreased hypersensitivity [57]. Additionally, neuropathic and inflammatory pain (hypersensitivity of the paw following inflammation) was abolished in *P2rx7* –/– mice, although acute pain responses remained unchanged [58]. Furthermore, the selective P2X7 antagonist A-740003 was effective at reducing neuropathic pain in rats [59].

P2X7 is also critically involved in inflammation; prolonged activation of P2X7 in immune cells such as macrophages leads to release of the pro-inflammatory cytokine interleukin-1β (IL-1β), activation of the inflammasome and, at times, cell death [60,61]. ATP-stimulated release of IL-1β was abolished in macrophages derived from *P2rx7*–/– mice [62], demonstrating the importance of P2X7 in inflammatory signalling.

Recently, a new range of P2X7-selective antagonists have been developed [63]. Of note, the P2X7 antagonist AZD9056 has provided promising results in Phase-II clinical trials for the treatment of rheumatoid arthritis [64]. These data raise hopes that new analgesic and anti-inflammatory treatments which target P2X receptors will soon be available.

## Box 2 Key outstanding questions

1. Can the location of the ATP binding site be confirmed? How does ATP bind?
2. How does ATP binding lead to channel opening and ion flux?
3. What is the structural basis for differential agonist and antagonist sensitivity?
4. What is the structural basis for differential receptor desensitization?
5. Which residues within the subunit–subunit contact regions are important for trimer assembly and stability?
6. What is the structure of the TM domain in a lipid bilayer?
7. What effect do the N-and C-termini of P2X receptors have on channel structure?
8. How do the structures of different P2X receptor subtypes differ from zebrafish P2X4.1? For example, *Dictyostelium* P2XA is only 16% identical in amino-acid sequence; does it have the same overall fold?
